# Acknowledgment of Reviewers

**DOI:** 10.2188/jea.JE20250597

**Published:** 2025-12-05

The following scientists assisted the journal by reviewing manuscripts during the period November 1, 2024 to October 31, 2025.

We gratefully acknowledge their critical evaluation and valuable assistance in selecting articles for publication.

**Table:** 

Abd El-Rahim, Ibrahim
Aizawa, Yuta
Amagasa, Shiho
Arakawa, Yuki
Arashiro, Takeshi
Arima, Yuzo
Ariyada, Kenichi
Asayama, Kei
Baba, Sachiko
Chen, Renjie
Choe, Seung-Ah
Choi, Ji-Yeob
Dao, Thi
Endoh, Kaori
Fu, Marcela
Fujihara, Kazuya
Fujii, Ryosuke
Fukushima, Noritoshi
Fukushima, Wakaba
Gallus, Silvano
Gastañaduy, Paul
Gilmour, Stuart
González-Marron, Adrián
Hanyuda, Akiko
Harada, Kazuhiro
Hatanaka, Sho
Hayashi, Takahito
Hirao, Susumu
Hirata, Aya
Hirayama, Atsushi
Hisamatsu, Takashi
Honda, Takanori
Hori, Ai
Hori, Daisuke
Horikawa, Chika
Horiuchi, Sayaka
Hosozawa, Mariko
Howden-Chapman, Philippa
Ide, Kazushige
Ihira, Hikaru
Iida, Miho
Imaizumi, Takahiro
Inoue, Kosuke
Ishida, Risa
Ishitsuka, Kazue
Ito, Yuri
Itoi, Shiori
Iwanaga, Mai
Kadowaki, Tomoka
Kamigaki, Taro
Kanehara, Rieko
Katanoda, Kota
Kataoka, Aoi
Kato, Tsuguhiko
Kawahara, Takuya
Kawakami, Ryoko
Kawamura, Chitose
Kazi, Ambreen
Kihara, Tomomi
Kim, Hyeon Chang
Kim, YoungWon
Kimura, Yasumi
Kishimoto, Kenji
Kishimoto, Yoshimi
Kitamura, Hiroko
Kitano, Naruki
Kiuchi, Sakura
Kiyohara, Kosuke
Kobayashi, Tohru
Kobayashi, Tomoaki
Kojima, Masahiro
Konishi, Takaaki
Koyanagi, Yuriko
Kuchiba, Aya
Kumwichar, Ponlagrit
Kuwahara, Keisuke
Lee, Mihye
Li-Mei, Chen
Li, Jiaqi
Ma, Enbo
Machida, Masaki
Mashimo, Sonoko
Masuda, Hiroya
Matsubara, Daisuke
Matsubara, Kousaku
Matsubara, Shigeki
Matsumoto, Naomi
Matsunaga, Takashi
Matsuo, Keitaro
Matsuyama, Yusuke
Matt, Georg E
Metoki, Hirohito
Michikawa, Takehiro
Mitsunami, Makiko
Mitsutake, Seigo
Miyamoto, Yoshihisa
Miyata, Jun
Mizumoto, Kenji
Morisaki, Naho
Murakami, Keiko
Muramatsu, Keiji
Nagai, Koutatsu
Nagayoshi, Mako
Nakagami, Gojiro
Nakagata, Takashi
Nakagomi, Atsushi
Nakao, Yoko
Nanri, Hinako
Nemoto, Yuta
Noda, Tetsuya
Nofuji, Yu
Noguchi, Taiji
Ohfuji, Satoko
Okada, Akira
Oono, Fumi
Otsuka, Rei
Oud, Lavi
Ozasa, Kotaro
Oze, Isao
Park, Bomi
Possenti, Irene
Romelli, Marco
Saijo, Yasuaki
Saito, Isao
Saito, Junko
Saito, Makoto
Sasai, Hiroyuki
Sasakabe, Tae
Sata, Mizuki
Sato, Kyoko
Sato, Shiho
Satoh, Michihiro
Sawada, Norie
Sekiyama, Makiko
Shibata, Ai
Shimokata, Hiroshi
Shinozaki, Tomohiro
Steen, Johan
Sugawara, Yumi
Sugiyama, Hiromi
Suzuki, Hideto
Tabuchi, Takahiro
Tada, Hayato
Taguri, Masataka
Taira, Kento
Tajima, Ryoko
Takaku, Reo
Takano, Yuta
Takehara, Kenji
Tanaka, Hirokazu
Tanaka, Junko
Tanaka, Kotone
Tanaka, Rina
Tanaka, Shigeho
Tanaka, Yoshio
Tatsuta, Nozomi
Terui, Toshihiro
Tian, Dechao
Tsuboya, Toru
Tsuji, Taishi
Tsujimoto, Takehiko
Ueda, Takuya
Ueta, Kayo
Ukawa, Shigekazu
Utada, Mai
Utsunomiya, Takeshi
Wakabayashi, Ryozo
Wakui, Tomoko
Wu, Lijun
Yamada, Masaaki
Yamada, Takuya
Yamaji, Taiki
Yamamoto, Seiichiro
Yamamoto, Takafumi
Yamana, Hayato
Yokomichi, Hiroshi
Yokoyama, Yuri
Zhou, Leilei

(in alphabetical order)

